# A signaling cascade including ARID1A, GADD45B and DUSP1 induces apoptosis and affects the cell cycle of germ cell cancers after romidepsin treatment

**DOI:** 10.18632/oncotarget.11647

**Published:** 2016-08-27

**Authors:** Daniel Nettersheim, Sina Jostes, Martin Fabry, Friedemann Honecker, Valerie Schumacher, Jutta Kirfel, Glen Kristiansen, Hubert Schorle

**Affiliations:** ^1^ Institute of Pathology, Department of Developmental Pathology, University Medical School, Bonn, Germany; ^2^ Tumor- and Breast Center ZeTuP Silberturm, St. Gallen, Switzerland; ^3^ Harvard Medical School, Department of Pediatrics, Boston, Massachusetts, USA; ^4^ Institute of Pathology, University Medical School, Bonn, Germany

**Keywords:** histone deactylase inhibitor, romidepsin, germ cell cancer, therapy, ARID1A

## Abstract

In Western countries, the incidence of testicular germ cell cancers (GCC) is steadily rising over the last decades. Mostly, men between 20 and 40 years of age are affected. In general, patients suffering from GCCs are treated by orchiectomy and radio- or chemotherapy. Due to resistance mechanisms, intolerance to the therapy or denial of chemo- / radiotherapy by the patients, GCCs are still a lethal threat, highlighting the need for alternative treatment strategies.

In this study, we revealed that germ cell cancer cell lines are highly sensitive to the histone deacetylase inhibitor romidepsin *in vitro* and *in vivo*, highlighting romidepsin as a potential therapeutic option for GCC patients.

Romidepsin-mediated inhibition of histone deacetylases led to disturbances of the chromatin landscape. This resulted in locus-specific histone-hyper- or hypoacetylation. We found that hypoacetylation at the *ARID1A* promotor caused repression of the SWI/SNF-complex member *ARID1A*. In consequence, this resulted in upregulation of the stress-sensors and apoptosis-regulators *GADD45B*, *DUSP1* and *CDKN1A*. RNAi-driven knock down of *ARID1A* mimicked in parts the effects of romidepsin, while CRISPR/Cas9-mediated deletion of *GADD45B* attenuated the romidepsin-provoked induction of apoptosis and cell cycle alterations.

We propose a signaling cascade involving *ARID1A*, *GADD45B* and *DUSP1* as mediators of the romidepsin effects in GCC cells.

## INTRODUCTION

Testicular type II germ cell cancers arise from a common precursor lesion, termed germ cell neoplasia *in situ* (GCNIS) [1–3]. GCNIS cells are the result of a defective germ cell development, where a primordial germ cell (PGC) is thought to suffer from genetic aberrations leading to a developmental arrest [2, 4]. GCNIS cells eventually differentiate into the invasive type II germ cell cancers (GCCs), which are subdivided into seminomas and non-seminomas [2]. Seminomas are highly similar to GCNIS and PGCs regarding gene expression and histology [2]. Contrarily, the stem cell population of the non-seminomas, the embryonal carcinoma (EC) shows features of totipotency and is therefore able to differentiate into all three germ layers (teratomas) and extraembryonic tissues (yolk-sac tumors, choriocarcinomas).

Familial predisposition, environmental parameters like exposure towards fertilizers, fine dust, endocrine disruptors and hormones are discussed as risk factors for development of GCCs [5]. Additionally, presence of the testicular dysgenesis syndrome (cryptorchidism, azoospermia and testicular atrophy) increases the risk for GCC development [6, 7].

Generally, GCCs are treated by orchiectomy and depending on stage with chemo- or radiotherapy in addition. Early stage seminomas are very radiosensitive. Thus, stage I - IIb seminomas are treated by radiotherapy, whereas non-seminomas are treated with chemotherapy. More advanced stages of seminoma or patients that do not tolerate radiotherapy also receive chemotherapy. Although most GCCs are sensitive towards a cisplatin-based therapy, approximately 20 - 50% of patients with metastatic disease cannot be cured by standard chemotherapy due to resistance mechanisms [8]. Thus, there is a strong need for new therapeutic options to treat cisplatin-resistant disease. In this study, we treated GCC lines with the histone deacetylase inhibitor (HDI) romidepsin (ISTODAX, FK228, FR901228) to elaborate on the molecular mechanism and to address the question whether it is a therapeutic option for GCCs.

## RESULTS

We reported previously that treatment of seminoma-like TCam-2 cells with romidepsin rapidly induced apoptosis [9]. Based on this initial finding, we asked if romidepsin might also be toxic to other GCC cell lines. Thus, in this study we analyzed its molecular mode of action *in vitro* and *in vivo* and elaborated on the potential of romidepsin as a new therapeutic for GCCs.

We utilized GCC cell lines and corresponding cisplatin-resistant subclones. The cell line TCam-2 was used as a proxy for a seminoma, while the three cell lines 2102EP, NCCIT and NT2/D1 were derived from ECs and the two cell lines JAR and JEG-3 resemble a choriocarcinoma in culture [10–14]. As controls we included human primary fibroblasts (MPAF, ARZ, EMF) and the Sertoli cell line (FS1) [15].

### Romidepsin efficiently kills GCC cells *in vitro* and *in vivo*

First, we treated seminoma-like TCam-2, the EC cell lines 2102EP and NCCIT as well as the choriocarcinoma-like JAR and JEG-3 cells with various concentrations (1 - 80 nM) of romidepsin for 24h and measured viability by a XTT assay (Supplementary Figure S1A). For comparison, cells were also treated with other HDIs (TSA, VPA, SAHA) and cisplatin. Romidepsin reduced viability starting from ≥ 5 nM, while VPA, SAHA and cisplatin had no detectable effect on viability at concentrations applied (Supplementary Figure S1A). Besides romidepsin, only TSA reduced viability of analyzed cell lines, but at much higher doses compared to romidepsin.

**Figure 1:**
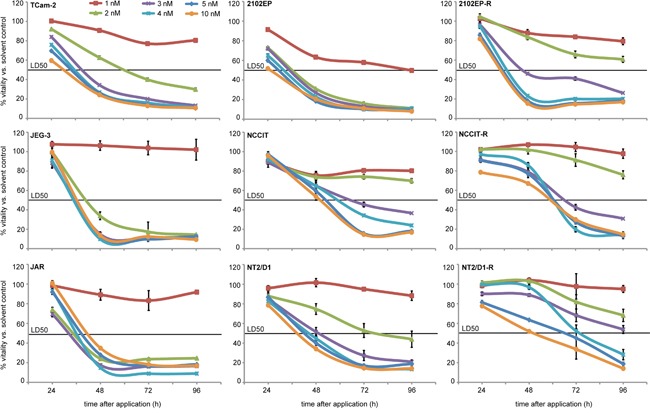
XTT assays of indicated GCC cell lines 24 - 96h after application of 1 - 10 nM romidepsin Standard deviation is given above each data point.

To determine the minimal effective concentration of romidepsin, we treated GCC cell lines once with 1 - 10 nM romidepsin and measured the effects on cell viability by XTT assays for 96h (Figure 1). While 1 nM had negligible effects on cell viability, a single application ≥ 2 nM strongly reduced viability in a dose-, time- and cell line-dependent manner within 4 days (Figure 1). These analyses demonstrate that GCC lines are highly susceptible to romidepsin treatment.

**Figure 2:**
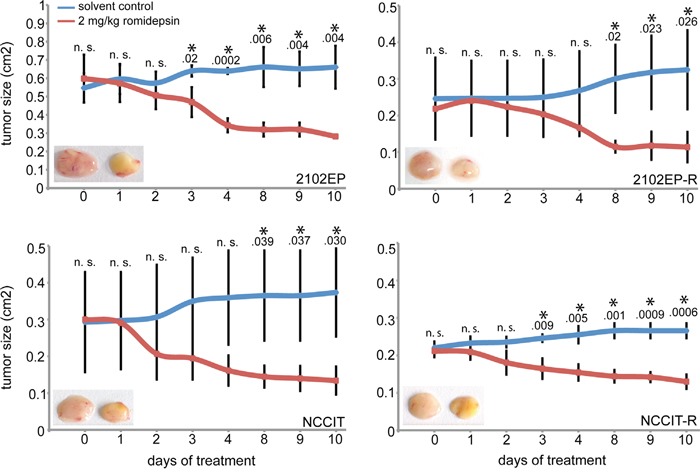
Measurement of the tumor burden during treatment of xenografted 2102EP(-R) and NCCIT(-R) cells with 2.5 mg/kg romidepsin or the solvent for 10 days Inlay: photos of tumors of solvent (left) and romidepsin (right) treated mice after 10 days. n. s. = not significant, p-value > 0.05; asterisk = significant, p-value < 0.05.

Next, we analyzed whether romidepsin kills GCC cells in an *in vivo* tumor model. To mimic GCCs, we xenografted 2102EP, 2102EP-R, NCCIT and NCCIT-R cells into the flank of nude mice and allowed tumors to grow for two weeks (-R = cisplatin-resistant subclone). Afterwards, we applied romidepsin (2 mg/kg) intravenously three times a week and monitored tumor growth for 10 days. Lately after 7 days, tumor sizes were significantly reduced in romidepsin treated mice compared to the control mice (Figure 2). We confirmed induction of apoptosis by detection of PARP cleavage in romidepsin treated mice bearing 2102EP-R and NCCIT-R tumors (Supplementary Figure S1B). In conclusion, romidepsin efficiently kills tumor cells *in vivo* by inducing apoptosis.

### GCC cells mainly utilize HDAC1 for histone deacetylation

Next, we were interested in alterations of molecular mechanisms induced by romidepsin in GCCs. HDIs like Romidepsin inhibit histone deacetylases (HDACs). Re-analyzing an expression microarray of GCC tissues published in a previous study [12] and a qRT-PCR analysis of GCC cell lines revealed that *HDAC1* is highly expressed in all GCCs, GCC cell lines, human fibroblasts (ARZ, MPAF) and the Sertoli cell line (FS1) (Supplementary Figure S1C, S1D). All other analyzed *HDACs* showed a lower expression compared to *HDAC1* in all analyzed GCC samples (Supplementary Figure S1C, S1D), indicating that GCCs might mainly utilize HDAC1 for histone deacetylation.

### Romidepsin causes hyperacetylation of histones H3 and H4

Since inhibition of HDACs should lead to histone hyperacetylation, we analyzed the pan-acetylation status of histones 3 and 4 (pan-H3ac / -H4ac) 2 - 16h after 10 nM romidepsin treatment of GCC cell lines, fibroblasts and the Sertoli cell line FS1. As shown by western blotting, within 2 - 16h after treatment H3 and H4 became hyperacetylated in all samples analyzed (Supplementary Figure S2A, S2B). In parallel, an ELISA-based quantification method revealed strong reduction of HDAC activity in protein lysates of TCam-2 and NCCIT cells 4 - 16h after romidepsin application (Supplementary Figure S2C). So, romidepsin reduced the activity of HDACs, leading to pan-H3 / H4 hyperacetylation.

### Romidepsin induces apoptosis and affects the cell cycle *in vitro*

**Figure 3:**
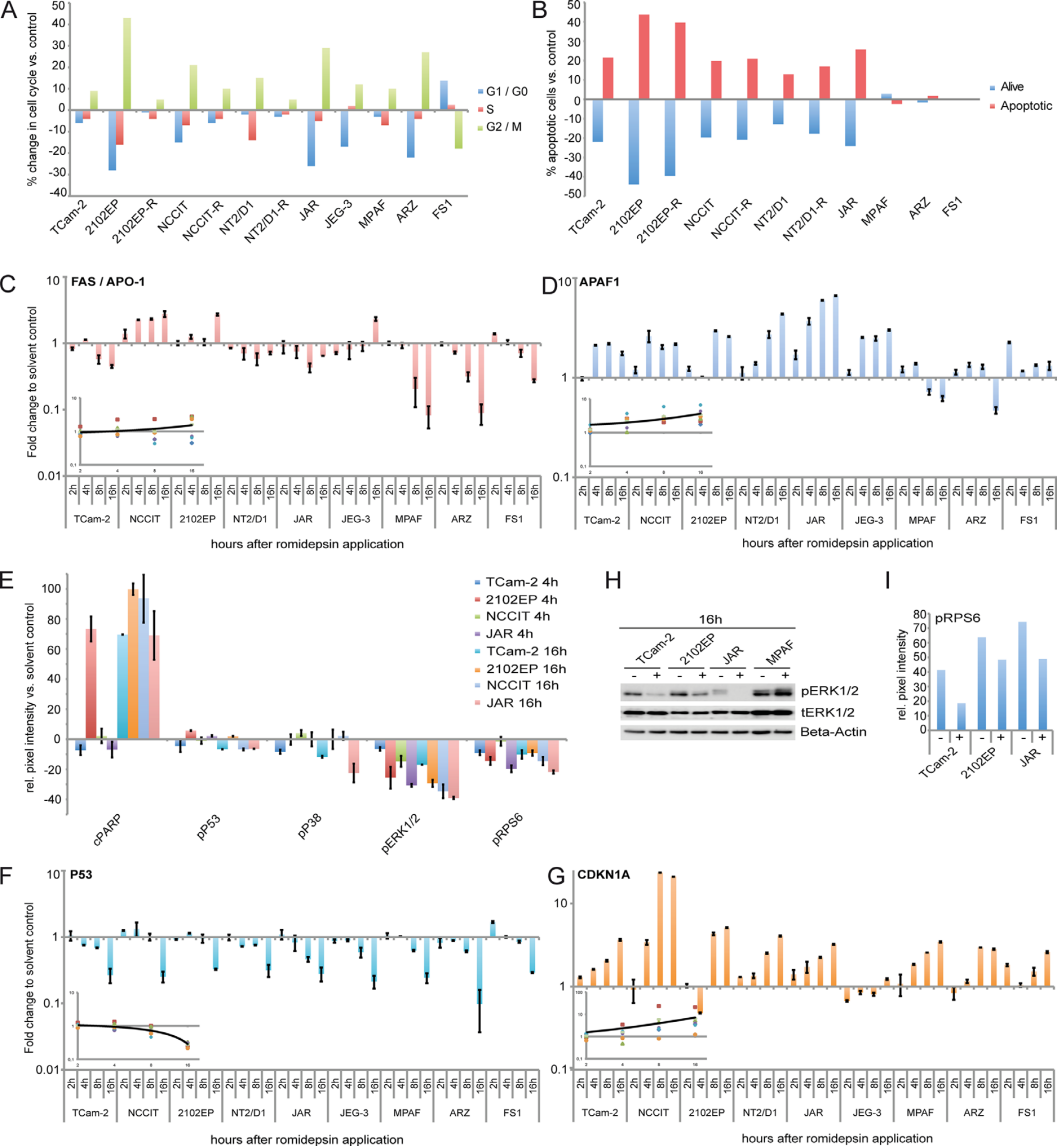
**A, B.** FACS-based cell cycle (A) and apoptosis (B) analysis of GCC cell lines, fibroblasts and FS1 cells 16 / 24h after 10 nM romidepsin application. **C, D.** qRT-PCR analysis of *FAS* / *APO-1* and *APAF1* in GCC cell lines, fibroblasts and FS1 cells 2 - 16h after 10 nM romidepsin treatment. Inlays: averaged expression changes over time (GCC cell lines only). **E.** PathScan analysis of indicated signaling molecules in GCC cell lines treated with 10 nM romidepsin for 4 and 16h. **F, G.** qRT-PCR analysis of *P53* and *CDKN1A* in GCC cell lines, fibroblasts and FS1 cells 2 - 16h after 10 nM romidepsin treatment. Inlays: averaged expression changes over time (GCC cell lines only). **H.** Western blot analysis of MAPK signaling factors in GCC cells and fibroblasts 16h after 10 nM romidepsin treatment. **I.** Densiometric evaluation of western blot data (Supplementary Figure S3G) of pRPS6 in GCC cell lines 16h after 10 nM romidepsin treatment.

Our XTT assays demonstrated that GCC cell lines display reduced proliferation / viability rates after romidepsin application. A PI-FACS analysis 16h after romidepsin treatment found a strong enrichment of GCC and fibroblast (MPAF, ARZ) cells in G2/M-phase of the cell cycle, while FS1 cells enriched in the G1/G0-phase (Figure 3A). Thus, GCC cell lines enrich in G2/M-phase of the cell cycle in response to romidepsin, which might be indicative of a G2-arrest.

Apoptosis was detected by a FACS-based AnnexinV / 7AAD analysis. Induction of apoptosis was seen in GCC cells treated with romidepsin (TCam-2, 2102EP(-R), NCCIT(-R), NT2/D1(-R) and JAR), while no apoptosis was observed in fibroblasts or the Sertoli cell line (Figure 3B). *FAS* and *APAF1* are markers for the death-receptor and intrinsic mitochondrial apoptosis pathway, respectively. *FAS* was only slightly deregulated in GCC cell lines, while *APAF1* was strongly upregulated in all GCC cell lines after romidepsin application (Figure 3C, 3D). This suggests that in response to romidepsin all GCC cells mainly induce apoptosis via the intrinsic mitochondrial pathway. Importantly, a strong downregulation of *FAS* and a nearly unchanged *APAF1* expression was found in all control cells (MPAF, ARZ, FS1), matching the observation that these cells do not initiate apoptosis in response to the concentrations of romidepsin, which are cytotoxic for GCC cell lines (Figure 3B, 3C, 3D).

### Alterations in signaling pathways in response to romidepsin

We performed a PathScan array, which detects the phosphorylation or cleavage status of 18 signaling molecules to identify signaling pathways activated by romidepsin (Supplementary Figure S3A). In all cell lines analyzed (TCam-2, 2102EP, NCCIT, JAR), we found a strong induction of PARP cleavage (Asp214) 16h after romidepsin application, further confirming induction of apoptosis (Figure 3E). By western blotting we verified cleavage of PARP in romidepsin treated TCam-2 cells, while no cleavage was found in fibroblasts, further suggesting that romidepsin does not induce apoptosis in fibroblasts (Supplementary Figure S3B).

Additionally, we detected nearly unchanged phosphorylation (p) of P53 (Ser15) after romidepsin treatment (Figure 3E). Further to very low levels of pP53 (Figure 3E), we detected downregulation of *P53* expression in all analyzed cell lines, while the *P53* downstream effector *CDKN1A* was strongly upregulated (except in JEG-3) (Figure 3F, 3G). CDKN1A upregulation was verified by western blotting in TCam-2, JAR and MPAF (Supplementary Figure S3C). By qRT-PCR analysis, we confirmed that the cisplatin-resistant GCC cell lines also upregulated *CDKN1A*, while *P53* was downregulated (2102EP-R) or remained unchanged (NCCIT-R, NT2/D1-R) (Supplementary Figure S3D). These findings confirm previous findings demonstrating that GCC cell lines induced *CDKN1A* in a P53-independent manner [16]. In P53-deficient human colon cancer cells *CDKN1A* induction is associated with G2/M-phase arrest [17]. Thus, upregulation of *CDKN1A* might contribute to enrichment of the cells in G2/M-phase after romidepsin application.

We found strongly reduced pERK1/2 (Thr202/Tyr204) levels in all GCC cells by the PathScan array (Figure 3E) and confirmed this finding by western blot analysis of TCam-2 and 2102EP cells (Figure 3F). Additionally, all analyzed cell lines showed reduced phosphorylation of the ERK1/2 signaling downstream target ribosomal protein S6 (RPS6; Ser235/Ser236) (Figure 3E, 3G; Supplementary Figure S3B). Decreased pRPS6 is associated with growth arrest. Thus, romidepsin treatment leads to a diminished pERK1/2 activity, resulting in reduced pRPS6 levels, which might contribute to the alterations in the cell cycle, i. e. enrichment in G2/M-phase.

### Dynamics of histone acetylation and gene expression in response to romidepsin

Since romidepsin inhibits HDACs, leading to histone hyperacetlyation, we performed ChIP-seq. against pan-H3ac in romidepsin / solvent treated TCam-2 to analyze the changes in detail. Pooled input DNA was used as control. In total, 8907 loci showed an at least 2-fold change in pan-H3ac occupation upon treatment with romidepsin compared to the solvent control (881 loci increased in pan-H3ac, 8026 loci decreased in pan-H3ac) (Supplementary Data S1A). These results demonstrate that a large number of loci seem to escape the global hyperacetylation induced by chromatin-wide inhibition of HDACs by romidepsin.

To analyze, whether the changes in acetylation caused by romidepsin correspond to changes in gene expression we next performed an expression microarray analysis of TCam-2 cells treated with 10 nM romidepsin for 8 and 16h (Supplementary Data S1B, S1C). Surprisingly, only 197 genes were upregulated and 125 genes were downregulated (≥ 2-fold) 16h after romidepsin application (Supplementary Data S1C). 16 of the 197 upregulated genes showed an increase in H3ac upon romidepsin treatment, while 84 of the 125 downregulated genes became hypoacetylated (Supplementary Data S1A). These findings suggest that the expression of a small fraction of genes is affected by romidepsin-mediated HDAC inhibition.

### Genes commonly deregulated upon romidepsin treatment in GCCs

**Figure 4:**
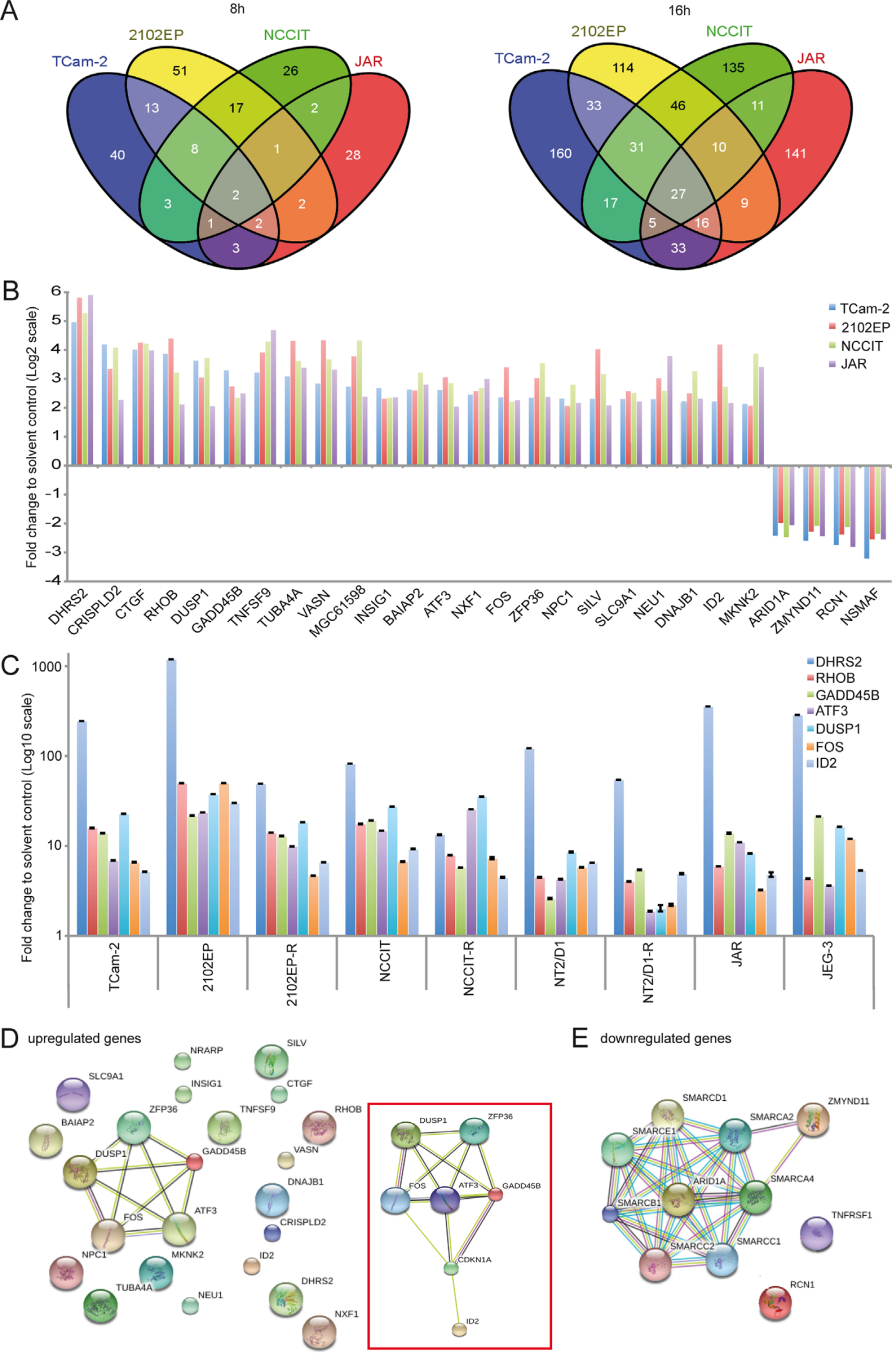
**A.** Venn diagrams of commonly expressed genes between GCC cell lines 8 and 16h after romidepsin treatment as found by expression microarray analysis. **B.** Expression levels of the 27 commonly deregulated genes (23 upregulated, 4 downregulated) in GCC cell lines. **C.** qRT-PCR verification of selected deregulations in romidepsin treated (16h) GCC cell lines. **D, E.** STRING protein-protein-interaction prediction of the 23 upregulated genes (D) and 4 downregulated genes (E). An extended network, including *CDKN1A* is illustrated in the red box (D).

To identify genes commonly deregulated in response to romidepsin, we extended our expression microarray analysis to 2102EP, NCCIT and JAR cells treated with 10 nM romidepsin for 8 and 16h (Supplementary Data S1B, S1C). In general, the number of deregulated genes increased from 8 to 16h after treatment and more genes were upregulated than downregulated (Supplementary Figure S4A). A Venn diagram (Figure 4A) shows that 2 genes (*DHRS2*, *GADD45B*) were commonly upregulated between the GCC cells 8h after romidepsin application, while 27 genes were deregulated after 16h (23 upregulated, 4 downregulated) (fold change (FC) ≥ Log_2_2) (Figure 4A, 4B; Supplementary Data S1D).

From the 23 upregulated genes, four genes (*BAIAP2*, *DNAJB1*, *MKNK2, SLC9A1)* showed increased H3ac levels upon romidepsin treatment of TCam-2 cells (Figure 4B; Supplementary Data S1A). Additionally, two of the four downregulated genes (*ARID1A*, *ZMYND11*) showed a decrease of H3ac in romidepsin treated TCam-2 (Figure 4B; Supplementary Data S1C, S1D).

As already demonstrated, *CDKN1A* was upregulated in all analyzed cell lines (FC ≥ Log_2_1,5 ≤ Log_2_2) (Supplementary Data S1E). We further verified upregulation of *DHRS2*, *RHOB*, *GADD45B*, *ATF3*, *DUSP1*, *FOS* and *ID2* in GCC cell lines (Figure 4C). By western blotting, we verified upregulation of GADD45B in 2102EP, NCCIT and JAR cells as well as upregulation of ID2 in 2102EP-R, NCCIT-R and NT2/D1-R (Supplementary Figure S3E).

A STRING analysis of the 23 upregulated genes predicted interaction of the stress-sensors DUSP1, GADD45B, ATF3 as well as the immediate early gene FOS and the ring finger protein ZFP36, suggesting that this network mediates the romidepsin-provoked stress- and apoptosis-response (Figure 4D). When manually including CDKN1A to the dataset, the interaction network was extended to CDKN1A and ID2 (Figure 4D, inlay). H3ac levels across the gene bodies of these romidepsin-response genes (*ATF3*, *DUSP1*, *FOS*, *GADD45B*, *ID2*, *ZFP36, CDKN1A)* were already high in the solvent control and did not increase further under romidepsin treatment of TCam-2 cells (Supplementary Figure S3F). Thus, these data show that upregulation of these genes occurs independent of euchromatin formation.

On the other hand, STRING analysis of downregulated genes predicted interaction of ARID1A and ZMYND11 with members of the ATP-dependent SWI/SNF-chromatin-remodeling-complex (SMARCs) (Figure 4E). As mentioned before, in TCam-2 cells downregulation of *ARID1A* correlates to decreasing H3ac levels at the promotor under romidepsin treatment (Supplementary Data S1A). So downregulation of *ARID1A* seems to be the result of heterochromatin formation.

Upon romidepsin application, the EC cell lines 2102EP and NCCIT cells tended to downregulate EC-, self-renewal- and pluripotency-associated genes *NANOG*, *OCT3/4*, *SOX2*, *DPPA3*, *DPPA4*, *GDF3*, *LIN28A*, *ZFP42*, *SALL4*, *PRDM14*, *ZIC3*, *UTF1*, *DNMT3B*, *BCAT1*, *SPRY4*, *YAP1* and *NODAL* (only 2102EP) (Supplementary Data S1D). Choriocarcinoma-like JAR cells downregulated *DPPA3*, *LIN28*, *ZFP42*, *SALL4*, *DNMT3L* and *YAP1*. TCam-2 cells showed downregulation of *NANOG*, *OCT3*/*4*, *DPPA4*, *GDF3*, *SPRY4*, *YAP1*, *UTF1* as well as *LIN28* and further downregulated the seminoma-associated genes *SOX17*, *IGF1* and *PRDM1* (Supplementary Data S1D). In TCam-2, downregulation of *IGF1*, *DPPA4*, *SPRY4*, *YAP1* and *ZSCAN10* correlated to decreasing H3ac levels at corresponding genomic loci, suggesting that expression of these factors might be silenced by heterochromatin formation (Supplementary Data S1A). These findings demonstrate that romidepsin application results in downregulation of pluripotency-related genes and EC / seminoma markers in TCam-2 and non-seminomatous GCC cells.

To measure the genome-wide effects of romidepsin on gene expression of our control cells, we performed a microarray analysis of fibroblasts and the Sertoli cell line FS1 8 and 16h after 10 nM romidepsin treatment. In general, control cells seemed less affected by a romidepsin treatment since only little changes in gene deregulation could be detected (Supplementary Figure S4A vs. S4B). We found significant deregulation of 12 (8h) and 47 (16h) genes in both fibroblast cultures (FC ≥ Log_2_2) (Supplementary Figure S4C, Supplementary Data S1F). We detected a common deregulation of 3 genes after 8h and 37 genes after 16h between fibroblasts and FS1 cells (Supplementary Figure S4C, Supplementary Data S1G). A comparison of the genes deregulated in fibroblasts and FS1 to the set of genes deregulated in GCC cell lines revealed a common upregulation of 4 genes (*DHRS2*, *RHOB*, *CRISPLD2*, *BAIAP2*) (Supplementary Figure S4C, Supplementary Data S1H). STRING analysis however could not predict any interaction between those 4 genes (Supplementary Data S1H). We verified upregulation of *DHRS2* and *RHOB* expression in fibroblasts, FS1 and GCC cells by qRT-PCR analysis (Supplementary Figure S4D).

### ARID1A is an important mediator of the romidepsin response in GCC cell lines

**Figure 5:**
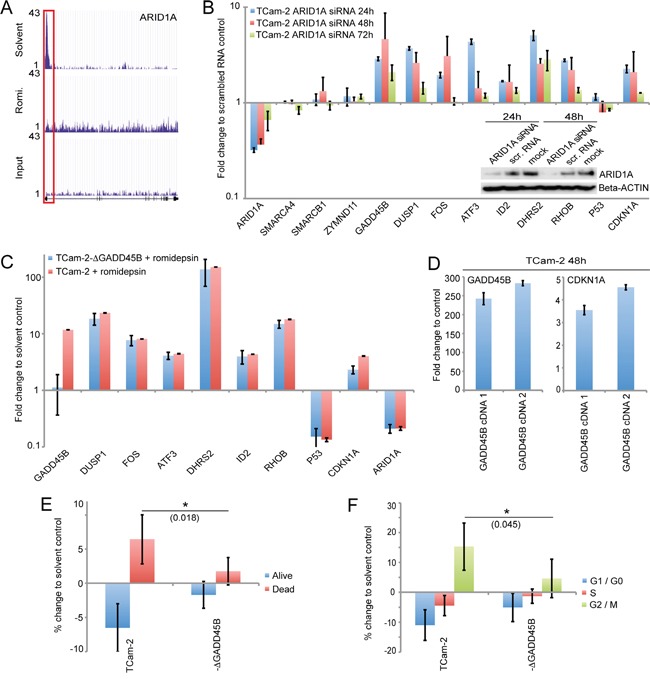
**A.** H3ac levels across the *ARID1A* gene as illustrated by the ‘UCSC Genome Browser’. The red box highlights the promotor region. **B.** qRT-PCR and western blot analysis (inlay) of indicated genes 24 - 72h after *ARID1A* siRNA transfection of TCam-2 cells. **C.** qRT-PCR analysis of indicated genes in parental and *GADD45B*-deficient TCam-2 cells 16h after treatment with romidepsin. Samples were normalized to corresponding solvent controls. **D.** qRT-PCR analysis of *GADD45B* and *CDKN1A* in TCam-2 cells transiently overexpressing *GADD45B* cDNA (1: 0.75 μg plasmid transfected, 2: 1 μg plasmid transfected). **E, F.** Annexin / 7AAD- (E) and PI-FACS (F) analysis of apoptosis and cell cycle distribution 16h after romidepsin treatment of TCam-2 and TCam-2-ΔGADD45B (−ΔGADD45B) cells. p-values were calculated by two-tailed Student's t-test and are given above the bars (* = significant; p-value < 0.05).

Our microarray analysis demonstrated that *ARID1A*, a member of the chromatin-remodelling SWI/SNF-complex is strongly downregulated upon romidepsin treatment of GCC cells. Additionally, the ChIP-seq analysis suggested that a decrease in acetylation of the chromatin covering the *ARID1A* promotor might repress *ARID1A* expression (Figure 5A, red box). The SWI/SNF-complex is involved in induction of cellular senescence and apoptosis as well as in oncogenesis and is required for transcriptional activation or repression of genes by chromatin-remodelling [18]. Thus, downregulation of the SWI/SNF-complex member *ARID1A* might influence chromatin-remodeling processes, resulting in altered transcription and DNA synthesis rates. So we analyzed the role of ARID1A in the molecular cascade of the romidepsin response in more detail.

First, we knocked down *ARID1A* by siRNA in TCam-2 cells and analyzed the expression of factors identified in this study. We achieved a strong *ARID1A* knock down 24 and 48h after transfection (ca. 70%), while 72h post transfection, *ARID1A* mRNA levels recovered to 35% (Figure 4B). Reduction of ARID1A protein levels was verified by western blot analysis (Figure 4B). 24 - 48h after knockdown, we detected upregulation of *GADD45B*, *DUSP1*, *FOS*, *ATF3*, *DHRS2*, *RHOB, ID2* and *CDKN1A*, while levels of *ZMYND11 SMARCA4* and *SMARCB1* remained unchanged (Figure 5B). After 72h expression levels of these factors began to normalize, correlating to recovering *ARID1A* mRNA levels.

We confirmed these results by transducing TCam-2 cells with four different *ARID1A* shRNA constructs and measured changes in gene expression after eight days (Supplementary Figure S5A). Efficient downregulation of *ARID1A* was demonstrated by qRT-PCR and western blot analysis, respectively (Supplementary Figure S5A). Again, we found upregulation of *GADD45B*, *DUSP1*, *FOS*, *ATF3*, *DHRS2*, *RHOB*, *ID2* and *CDKN1A* as well as unchanged *P53* expression. In conclusion, downregulation of *ARID1A* during a romidepsin treatment contributes to upregulation of stress, cell cycle and apoptosis regulators.

Of note, pluripotency in murine embryonic stem cells is maintained by SWI/SNF-complex activity and its component ARID1A contributes to proper expression of OCT3/4, SOX2 and UTF1 [19]. Vice versa, pluripotency factors, like NANOG, GDF3, OCT3/4 and SOX2 interact with SWI/SNF-complex members and influence higher order chromatin structure at pluripotency-associated gene promotors [20]. After romidepsin treatment, we detected downregulation of pluripotency factors in the EC lines 2012EP and NCCIT as well as the seminoma-like TCam-2 (Supplementary Data S1E). In TCam-2 cells, an *ARID1A* shRNA-mediated knock down resulted in downregulation of *NANOG*, *OCT3/4*, *LIN28* and *GDF3* expression and H3ac levels strongly decreased in pluripotency-associated gene promotors upon romidepsin application (Supplementary Figure S5B, S5C). Thus, we postulate that *ARID1A* supports the expression of pluripotency factors in GCC cell lines.

### GADD45B contributes to induction of apoptosis and affects the cell cycle

Upon cellular stress, *GADD45B* induces apoptosis and growth arrest [21]. *GADD45B* is upregulated in romidepsin treated GCC cells and after *ARID1A* knock down in TCam-2 cells (this study). So, does GADD45B contribute to induction of apoptosis and cell cycle alterations in response to romidepsin? Here, we established five *GADD45B*-deficient TCam-2 clones (TCam-2-ΔGADD45B1 - 5) by the gene-editing CRISPR/Cas9 technique (Supplementary Figure S5D). Three clones (1, 2, 4) displayed a 157 bp and a 518 bp band in a genotyping PCR, indicating that CRISPR/Cas9-mediated gene-editing resulted in a 902 bp and a 541 bp deletion, respectively. Two clones (3, 5) displayed a 541 bp deletion on both alleles. No band corresponding to the wildtype *GADD45B* locus was found in the TCam-2-ΔGADD45B clones. These data validate successful deletion of *GADD45B* in the TCam-2-ΔGADD45B clones.

A qRT-PCR analysis after treatment with romidepsin demonstrated induction of *GADD45B* in the parental TCam-2 cells and no expression in TCam-2-ΔGADD45B clones (Figure 5C). Interestingly, in TCam-2-ΔGADD45B cells upregulation of *CDKN1A* is only half the level compared to parental TCam-2 cells.

Further overexpression of *GADD45B* in TCam-2 cells by transfecting two different amounts of a *GADD45B* cDNA-coding plasmid (1: 0.75 mg, 2: 1 mg) resulted in a dose dependent upregulation of *CDKN1A* (Figure 5D). These data suggest that *GADD45B* contributes to the upregulation of *CDKN1A*. Notably, expression levels of the other stress sensors and apoptosis as well as cell cycle regulators remained unchanged between parental TCam-2 and TCam-2-ΔGADD45B clones, suggesting that *GADD45B* has no influence on the expression of these factors (Figure 5C).

We asked if *GADD45B*-deficiency attenuates romidepsin-induced apoptosis and cell cycle alterations. Indeed, an Annexin V / 7AAD-FACS analysis demonstrated that the percentage of apoptotic cells is significantly lower in TCam-2-ΔGADD45B clones 16h after romidepsin treatment (1.75% more compared to solvent control) than in parental TCam-2 cells (6.4% more compared to solvent control (Figure 5E). Additionally, 16h after romidepsin treatment a PI FACS demonstrated that only 4.6% of TCam-2-ΔGADD45B cells enriched in G2/M-phase of the cell cycle (compared to solvent control), while 15.3% of the parental TCam-2 enriched in G2/M-phase (compared to solvent control) (Figure 5F). Thus, *GADD45B* seems to contribute to induction of apoptosis and enrichment in G2/M-phase of the cell cycle in response to romidepsin. Parts of these effects might be mediated by CDKN1A.

### Deregulations in gene expression found *in vitro* are reflected *in vivo*

**Figure 6:**
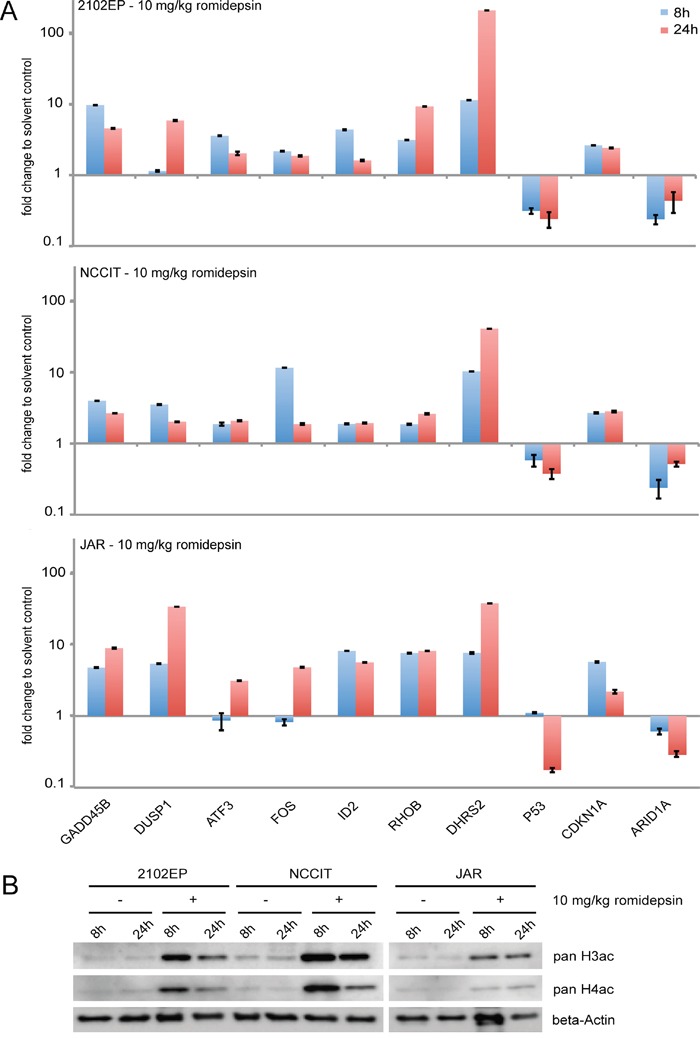
**A.** qRT-PCR analysis of 2102EP, NCCIT and JAR tumor tissues. Cells were xenografted for 2 weeks and then treated for 8 and 24h with 10 mg/kg romidepsin i. v. **B.** Western blot analysis of pan-H3ac / H4ac in 2102EP, NCCIT and JAR tumor tissues treated with 10 mg/kg romidepsin for 8 and 24h.

Finally we asked, if the results gathered in our *in vitro* experiments are also reflected *in vivo*. Thus, we xenografted 2102EP, NCCIT and JAR cells into the flank of nude mice and led tumors grow for two weeks. Afterwards, romidepsin (10 mg/kg) was injected intravenously and tumors were recovered after 8 and 24h. A qRT-PCR analysis demonstrates that the same genes were deregulated in expression as found *in vitro* (Figure 6A). In addition, histones H3 and H4 became hyperacetylated (Figure 6B). So, the effects caused by romidepsin are highly comparable *in vitro* and *in vivo* and conclusions drawn from our *in vitro* studies can reliably be transferred to the *in vivo* situation.

## DISCUSSION

In this study, we demonstrated that romidepsin is highly toxic at low concentrations to GCC cells *in vitro* and *in vivo*. Romidepsin led to a disturbed chromatin landscape, resulting in hyper- and hypoacetylation of the histones. H3-hypoacetylation at the *ARID1A* promotor leads to downregulation of *ARID1A* expression, resulting in upregulation of the stress-sensors *GADD45B* and *DUSP1*.

**Figure 7:**
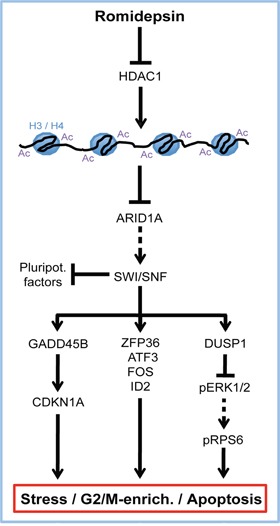
Model summarizing the molecular mode of action of romidepsin in GCCs Arrow = ‘activation / induction’, T-shaped arrow = ‘inhibition / repression’, dashed arrows = ‘abolished interaction’, Ac = acetylation, H3 / H4 = histones 3 and 4.

Based on our results, we propose a model of the molecular mechanisms of romidepsin in GCC cells (Figure 7). Downregulation of *ARID1A* in response to romidepsin induces expression of stress-sensors and apoptosis as well cell cycle regulators (*GADD45B*, *ATF3*, *FOS*, *ZFP36*, *DUSP1*, *ID2* and *CDKN1A*). We demonstrated that the stress-sensor *GADD45B* contributes to *CDKN1A* upregulation, induction of apoptosis and G2/M-phase enrichment. The immediate early gene and MAKP signaling inhibitor *DUSP1* is induced in response to environmental stress and romidepsin [22] (this study). We hypothesize that in response to romidepsin *DUSP1* mediated repression of the ERK1/2 signaling cascade leads to reduced phosphorylation of P90 ribosomal S6 kinase (RSK), which in consequence results in diminished pRPS6 levels [23]. Reduction of ERK1/2 signaling and pRBS5 levels are associated with growth arrest and apoptosis [23, 24]. These effects are summarized in our model and synergistically contribute to induction of stress, apoptosis and enrichment of romidepsin treated cells in the G2/M-phase of the cell cycle (Figure 7).

Importantly, the molecular effects found *in vitro*, like upregulation of stress- and apoptosis-regulators, cleavage of PARP and hyperacetylation of histones H3 and H4 were also found in tumors from xenografted GCC cell lines. Thus, we postulate that we molecular mode of action is highly similar *in vitro* and *in vivo*.

Our study demonstrates that romidepsin seems to be an interesting treatment option, especially for metastasized and / or cisplatin-resistant GCCs.

## MATERIALS AND METHODS

### Cell culture

All GCC cell lines used in this study were cultivated as described previously [14]. 2102EP-R, NCCIT-R and NT2/D1-R were cultivated as their corresponding parental cell lines. MPAF, ARZ and EMF (provided by Dr. Michael Peitz (Life & Brain, Department of Reconstructive Neurobiology, Bonn)) as well as FS1 (provided by Valerie Schumacher, [15]) were grown in DMEM medium (10% FCS (20% FS1), 1% Penicillin / Streptomycin, 200 mM L-Glutamine, 1x non-essential amino acids, 100 nM ß-Mercaptoethanol) at 37°C and 5% CO_2_.

### GCC and testis tissues

Seminoma (n = 4) and EC (n = 2) tissues as well as normal testis tissue were obtained from the Institute of Pathology (University Medical School, Bonn, Germany).

An internal review board allowed the use of these tissues in context of this study. Written permission to use the tissue for scientific purposes was obtained from the patients and was approved by the ‘ethics committee for clinical trials on humans and epidemiological research with patient-related data of the medical faculty of the Rheinischen- Friedrich-Wilhelms-University Bonn’. No personal patient data will be collected or stored in this study.

### HDI application

Romidepsin was dissolved in dimethyl sulfoxide (DMSO; AppliChem GmbH, Darmstadt, Germany) and diluted in phosphate buffered saline (PBS) to a stock solution of 4.62 mM. Romidepsin was provided by Gloucester Pharmaceuticals (Celgene, Signal Pharmaceuticals, LLC, San Diego, CA, USA; MTA ID #CC0488464). SAHA and TSA (both from Sigma-Aldrich, Taufkirchen, Germany) were diluted in DMSO to 94.5 mM and 500 μM, respectively. VPA (Sigma-Aldrich) was diluted in H_2_O (dest.) to a 301 μM stock solution and cisplatin (Sigma-Aldrich) was diluted in dimethylformamide (DMF; Merck, Darmstadt, Germany) to 41.65 mM.

### RNA and protein isolation

Total RNA and proteins were isolated as described previously [14]. Briefly, RNA was isolated by the RNAeasy mini kit (Qiagen, Hilden, Germany) and proteins by RIPA buffer.

### Western blot

Western blots were performed as described previously [14]. Beta-ACTIN was used as housekeeper and loading control. See Table 1 for antibody details.

**Table 1:** Antibodies used in this study

Antibody	Company	Clone / Order No.	Western Blot
ARID1A	Cell signaling	D2A8U / 12354	1:500
Beta-Actin	Sigma-Aldrich	AC-15	1:20000
CDKN1A	Santa Cruz	C-19 / sc-397	1:500
cleaved PARP	Abcam	ab4830	1:500
GADD45B	Sigma-Aldrich	AV48346	1:1000
H3 pan-acetylation	Active motif	39139	1:500
H4 pan-acetylation	Active motif	39243	1:750
ID2	Cell signaling	D39E8 / 3431	1:500
phospho-ERK1/2	Cell signaling	197G2 / 4377	1:1000
total-ERK1/2	Cell signaling	9102	1:5000

### Quantitative RT-PCR

Quantitative RT-PCR (qRT-PCR) was performed as published previously [14]. 500 ng of total RNA was used for first strand synthesis (FSS). *GAPDH* was used as housekeeping gene and for data normalization. See Table 2 for primer sequences.

**Table 2:** Oligonucleotides used in this study

Gene	Forward primer	Reverse primer	Tan	Cycles
APAF1	ACAATGCTCTACTACATGAA GGATATAAAGA	CACTGGAAGAAGAGACAACAGGAA	60°C	40
ARID1A	TCTTGCCCATCTGATCCATT	CCAACAAAGGAGCCACCAC	60°C	40
ATF3	AAGAACGAGAAGCAGCATTTGAT	TTCTGAGCCCGGACAATACAC	60°C	40
CDKN1A	CCTCATCCCGTGTTCTCCTTT	GTACCACCCAGCGGACAAGT	60°C	40
DHRS2	CTCCATGTAGGGCAGCAACT	GTAGGGAGCACTCTGGGGAC	60°C	40
DUSP1	GTACATCAAGTCCATCTGAC	GGTTCTTCTAGGAGTAGACA	60°C	40
FAS / APO-1	AGCTTGGTCTAGAGTGAAAA	GAGGCAGAATCATGAGATAT	60°C	40
FOS	GAGAGCTGGTAGTTAGTAGCATGTTGA	AATTCCAATAATGAACCCAATA GATTAGTTA	60°C	40
GADD45B	GTCGGCCAAGTTGATGAAT	CACGATGTTGATGTCGTTGT	60°C	40
GADD45B genotyp.	CCCGTCACTGATCCCTCTTT	CTACTGCGAAGAAAGCCGG	55°C	35
GAPDH	TGCCAAATATGATGACATCAAGAA	GGAGTGGGTGTCGCTGTTG	60°C	40
HDAC1	TAAATTCTTGCGCTCCATCC	AACAGGCCATCGAATACTGG	60°C	40
HDAC2	CGTGTAATGACGGTATCATTCC	ACCAGATAATGAGTCTGCACC	60°C	40
HDAC3	CTGTGTAACGCGAGCAGAAC	GCAAGGCTTCACCAAGAGTC	60°C	40
ID2	TCAGCCTGCATCACCAGAGA	CTGCAAGGACAGGATGCTGATA	60°C	40
P53	TTGCAATAGGTGTGCGTCAGA	AGTGCAGGCCAACTTGTTCAG	60°C	40
RHOB	GGGACAGAAGTGCTTCACCT	CGACGTCATTCTCATGTGCT	60°C	40
SMARCA4	CAGCATGCCAAGGATTTCAAG	CGATCCGCTCGTTCTCTTTC	60°C	40
SMARCB1	AACGTCAGCGGGTTCAAAT	GCCTTCACCTGGAACATGAA	60°C	40
ZMYND11	TTGTTAAACGTGCCATGACC	GCATGTGTGGAGACAGAGGA	60°C	40

Gene		shRNA sequence		
ARID1A sh1		5'-TCGCCTCTATGTGTCTGTGAAGGAGATTG-3'		
ARID1A sh2		5'-GCAAACTCAGCATCCAGGACAACAATGTG-3'		
ARID1A sh3		5'-CCTTGCTGTCTCAGCAGCCAATCAACTTA-3'		
ARID1A sh4		5'-CCATTTGTGGTGGACTGCTCAGATAACGT-3'		

Gene		gRNA sequence		
GADD45B gRNA 1		5'-CACAGTGGGGGTGTACGAGT-3'		
GADD45B gRNA 2		5'-ACAACGACATCAACATCGTG-3'		
GADD45B gRNA 3		5'-CTCTTCCAGGCGTCCGTGTG-3'		

### siRNA and cDNA transfection

Transfection of TCam-2 cells with siRNA or a cDNA-coding plasmid was performed as described previously [9]. Briefly, 1×10^5^ cells were seeded onto 6-well plates 24h before transfection. For transfection, FuGeneHD (Promega, Mannheim, Germany) was used at a ratio of 5: 1 (e.g. 5 μl FuGeneHD: 1 μg siRNA). *ARID1A* siRNAs were obtained from Santa Cruz Biotechnology as a pool of three different siRNAs (Santa Cruz, Heidelberg, Germany). Scrambled siRNA was used as negative control and for data normalization (AllStars Negative Control siRNA; Qiagen). siRNA experiments were performed in biological triplicates (n = 3). The *GADD45B* cDNA-coding plasmid was obtained from Origene (via Biocat, Heidelberg, Germany). The empty vector was used as negative control and for data normalization.

### shRNA transduction

For retroviral transduction of TCam-2 cells, four different *ARID1A* shRNAs encoded in the pGFP-V-RS vector were utilized (Origene, via BioCat, Heidelberg, Germany). The empty vector was used as negative control and for data normalization. See Table 2 for the sequences of the oligonucleotides used for generation of the *ARID1A* targeting shRNA-expressing plasmids. Retroviral particles were produced in 1.2×10^6^ HEK293 cells by transfecting 2 μg of the retroviral shRNA, 2 μg pCMV-gag-pol-plasmid and 220 ng pCMV-VSV-G-plasmid via the calcium phosphate method. The next day, medium was replaced by fresh medium. On day 3, the supernatant was harvested, sterile filtered and applied to the target cells. *ARID1A* shRNA expressing cells were selected by adding 0.5 mg/ml puromycin every second day for one week.

### Establishment of GADD45B-deficient TCam-2 cells by CRISPR/Cas9 gene-editing

Five different TCam-2-ΔGADD45B clones were generated as described previously [25]. Briefly, by using FuGeneHD (Promega) 1×10^5^ TCam-2 cells were transfected simultaneously with the pX330 vector encoding for three different guide RNAs (gRNA) directed against the *GADD45B* locus (Santa Cruz) and a GFP-coding plasmid. See Table 2 for gRNA sequences. Two days after the transfection, GFP-positive clones were manually picked and clonally expanded. Deletions within the coding sequence of *GADD45B* in each clone were detected by PCR (Supplementary Figure S5D). See Table 2 for primers used for genotyping.

### Measurement of HDAC activity

To measure the activity of HDACs we utilized the Epigentek ‘EpiQuik HDAC Activity / Inhibition Assay Kit’ (Epigentek, via Biocat). The procedure was performed as recommended in the manual. Whole protein lysates were prepared by NP-40 isolation buffer (150 mM sodium chloride, 1% NP-40, 50 mM Tris (pH 8)). 10 μg of protein was used for analysis.

### XTT assay

For the XTT-assay (2,3-bis-(2-methoxy-4-nitro-5-sulfophenyl)-2H-tetrazolium-5-carboxanilide), 24h before starting the experiment 5000 cells (8 x) were seeded in 100 μl standard growth medium without phenol red per well of a 96-well plate. The next day, different concentrations of HDIs or corresponding solvents were added to the cells. At the desired time points, 50 μl XTT (1 mg/ml) plus 1 μl PMS (1.25 mM) (both from Sigma-Aldrich) were added and absorbance was measured 4 h later in an ELISA reader (450 nm vs. 650 nm). Each time point / concentration was measured in four technical replicates.

### FACS-based propidium iodide and AnnexinV / 7AAD measurement

For propidium iodide (PI) FACS cell cycle analysis, 5×10^5^ cells were washed once in PBS, centrifuged for 5 min at 1000 rpm and fixed by re-suspending in 300 μl PBS + 700 μl ice cold 100% ethanol. After 1 - 2h of fixation at -20°C, cells were pelleted by centrifugation (5 min, 1000 rpm) and re-suspended in 1 ml DNA staining solution (PBS + 2 μl PI (1 mg/ml), + 20 μl RNAseA (10 mg/ml)). After 15 min of incubation at RT under constant agitation samples were analyzed by FACS. First, the single cell population was identififed in a forward versus side scatter plot. Duplets or clumbs and cell debris was excluded from analyses. Next, cell cycle distribution was detected by a forward scatter versus PI plot and illustrated as a histogram by plotting PI versus cell. For apoptosis measurement, the ‘AnnexinV Apoptosis Detection Kit I’ was used (BD Biosciences, Heidelberg, Germany). Early and late apoptotic as well as viable cell populations were identified by plotting PE AnnexinV versus 7-AAD. Analysis was performed according to the manual. For each measurement, three independent samples were pooled. For PI and AnnexinV FACS, in total 50000 cells were measured using the FACSCanto machine and analyzed by the FACSDiva Software (BD Biosciences).

### Illumina HT-12v4 expression microarray

The Illumina expression microarray analyses were performed exactly as published [14]. Briefly, RNA quality was checked in a BioAnalyzer 2100 (RNA 6000 nano lab chip) (Agilent Technologies, Waldbronn, Germany). Samples were processed on Illuminas human' HT-12v4' chips (San Diego, California, USA). Technical quality parameters such as hybridization, extension and specificity were evaluated using the ‘Genome Studio’ software. Expression values were quantile normalized using the ‘limma’-software-package (‘Linear Models for Microarray Data’, www.bioconductor.org). The microarray data set is publically available via GEO (ncbi.nlm.nih.gov/geo/) (GSE71239).

### Affymetrix expression microarray analysis of GCC tissues

The whole procedure and the microarray data have already been published [12]. The microarray data were re-analyzed in context of this study and includes normal testis tissue (n = 4), GCNIS (n = 3), seminomas (N = 4), ECs (n = 3), teratomas (n = 3) and mixed non seminomas (n = 4). All tumors were classified according to the WHO classification of tumors based on their histology and assessment of tumor or GCNIS amount.

### Chromatin-immunoprecipitation-followed-by-sequencing

For chromatin-immunoprecipitation-followed-by-sequencing (ChIP-seq) analysis, cells of interest were fixed by Formaldehyde solution (11% Formaldehyde, 0.1 M NaCl, 1 mM EDTA (pH 8), 50 mM HEPES (pH 7.9)) and further processed by Active Motif (Carlsbad, CA, USA), involving shearing of DNA by sonication, chromatin-immunoprecipitation, library generation and sequencing (NextSeq500, Illumina). Pooled input DNA of each sample including spike-in Drosophila DNA were used as controls and for normalization. The 75-nt sequence reads were mapped against the genome using BWA algorithm. Duplicate reads were removed. Only peaks that align with no more than 2 mismatches and map uniquely to the genome were used for further analysis. Intervals / peaks were identified by the MACS peak finding algorithm (cutoff p-value 1×10^−7^) including ENCODE blacklist filtering [26]. ChIP-seq data was visualized using the ‘UCSC Genome Browser’ (www.genome-euro.ucsc.edu). ChIP-seq data sets are publically available via GEO (GSE78262).

### Xenotransplantation of TCam-2 and 2102EP cells

Xenotransplantation was performed as described in a previous publication [14]. 1×10^7^ TCam-2 and 2102EP cells were re-suspended in 4°C cold 500 μl Matrigel (BD Biosciences) and injected into the right flank of nude mice using a pre-cooled 28G syringe (BD Biosiences). At the day of romidepsin application, the mice were weighted allowing for accurate calculation of the correct dosages. A romidepsin solution of desired concentration was prepared freshly. Nude mice were pre-warmed on a 37°C warm plate allowing for a more easy injection procedure due to enlarging of blood vessels. Afterwards, mice were fixed in a fixator and 100 μl of the romidepsin solution were injected intravenously into the vein of nude mice using a 28G syringe (BD Biosciences). For each experimental condition, three mice were analyzed. Animal experiments were conducted according to the German law of animal protection and in agreement with the approval of the local institutional animal care committees (Landesamt für Natur, Umwelt und Verbraucherschutz NRW (approval ID: AZ84-02.04.2013.A430)). The experiments were performed in accordance with the ‘International Guiding Principles for Biomedical Research Involving Animals’ as announced by the ‘Society for the Study of Reproduction’.

### STRING analysis

STRING protein-interaction predictions were performed online using default settings (www.string-db.org) [27].

## SUPPLEMENTARY FIGURES AND TABLE




