# Supplementary material for: A signaling cascade including ARID1A, GADD45B and DUSP1 induces apoptosis and affects the cell cycle of germ cell cancers after romidepsin treatment
Source: Oncotarget. 2016 Aug 27;7(46):74931–46. doi: 10.18632/oncotarget.11647 (PMC5342713; doi:10.18632/oncotarget.11647)
Supplement: Supplementary file 1 [file oncotarget-07-74931-s001.pdf]

A signaling cascade including ARID1A, GADD45B and DUSP1 induces apoptosis and affects the cell cycle of germ cell cancers after romidepsin treatment

SUPPLEMENTARY FIGURES AND TABLE

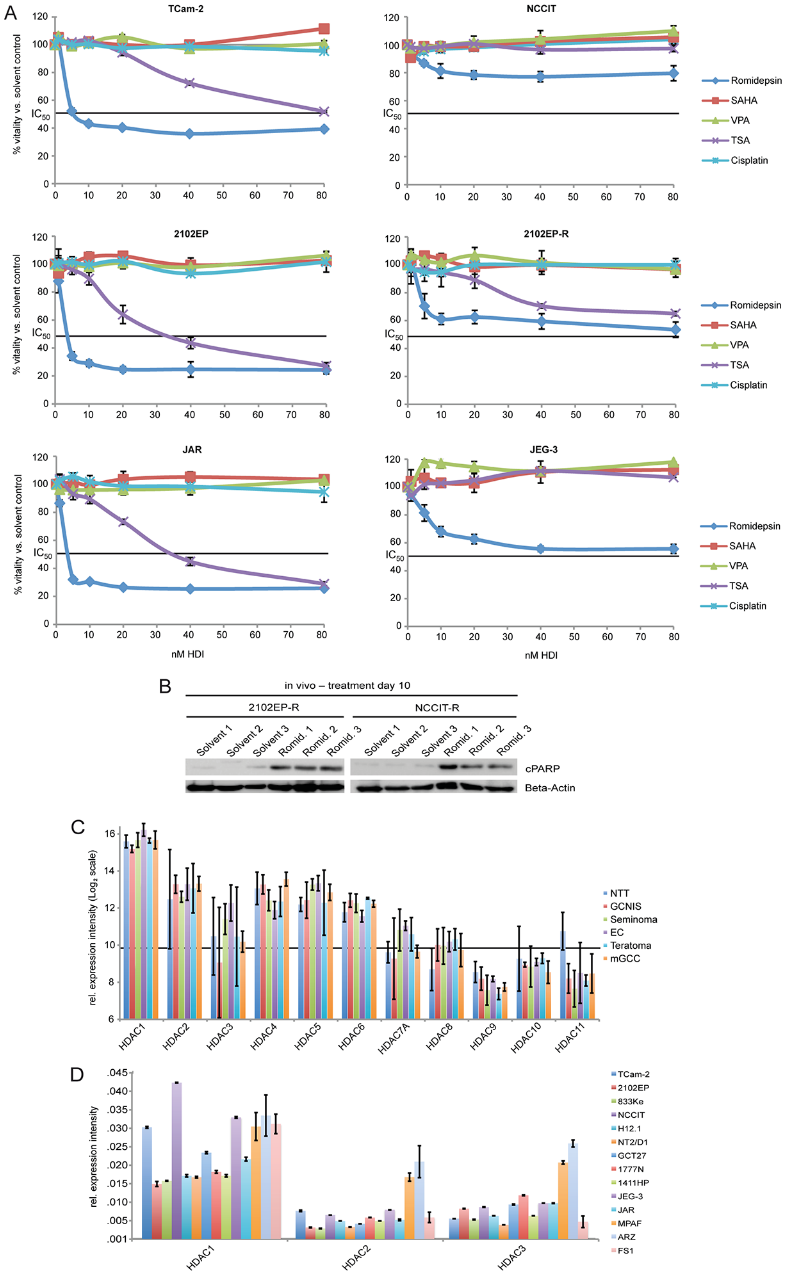

Supplementary Figure S1: A. XTT-assay of indicated GCC cell lines 24h after 1 - 80 nM romidepsin, SAHA, VPA, TSA or cisplatin application. **B.** Western Blot analysis of cleaved PARP in 2102EP-R and NCCIT-R tumor tissues 10 days after treatment with romidepsin or the solvent. **C.** *HDAC1 - 11* expression in normal testis tissue (NTT) and human GCC tissues found by an expression microarray analysis published previously [12]. Genes showing expression intensities below horizontal black line were considered as being not expressed. **D.** qRT-PCR analysis of *HDAC1 - 3* in indicated GCC cell lines, fibroblasts and FS1 cells. C) XTT-assay of 1 - 8 µM cisplatin-treated NCCIT and NCCIT-R cells.

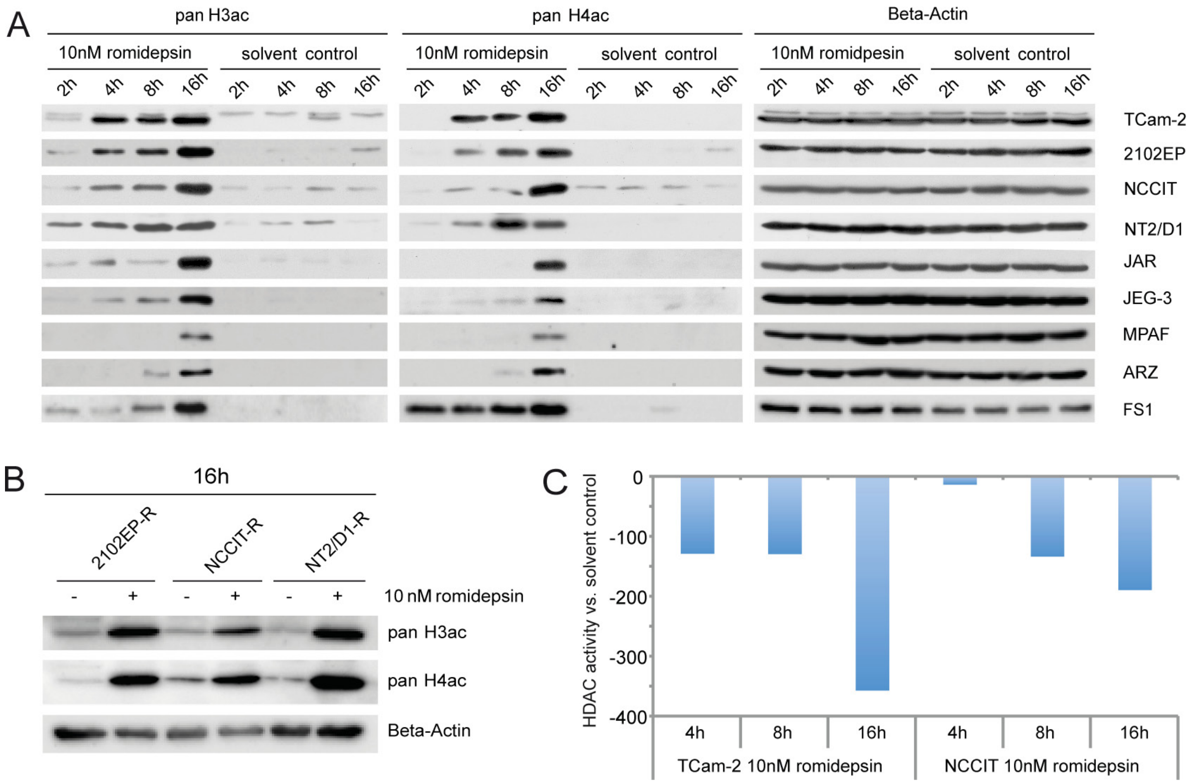

Supplementary Figure S2: A. Western blot analysis of pan H3ac / H4ac levels 2 - 16h after 10 nM romidepsin application in indicated GCC cell lines, fibroblasts and FS1 cells. **B.** Western blot analysis of pan H3ac / H4ac levels 16h after 5 nM romidepsin application in indicated cisplatin-resistant GCC cell lines. **C.** ELISA-based measurement of total HDAC activity in whole protein lysate (WPL) of TCam-2 and NCCIT cells 4 - 16h after 10 nM romidepsin application.

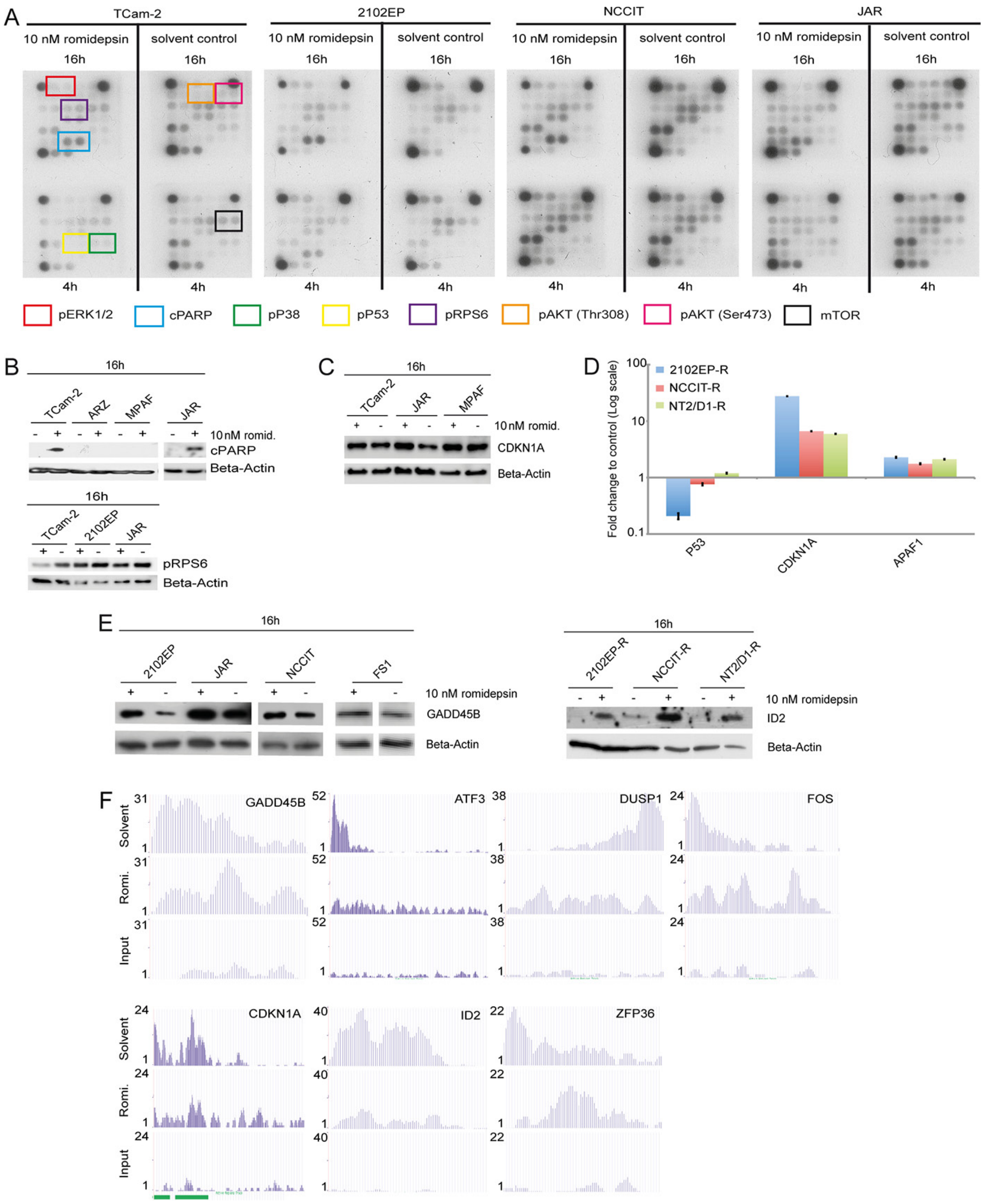

Supplementary Figure S3: A. PathScan raw data. Indicated cell lines were treated with 10 nM romidepsin for 4 and 16h. **B.** Western blot analysis of PARP cleavage in indiacted GCC cell lines and fibroblasts 16h after romidepsin treatment. **C.** Western blot analysis of CDKN1A 16h after 10 nM romidepsin application in indicated GCC cell lines and fibroblasts. **D.** qRT-PCR analysis of indicated genes in cisplatin-resistant GCC cell lines 16h after 5 nM romidepsin treatment. **E.** Western blot analysis of GADD45B and ID2 expression 16h after 5 nM romidepsin application in indicated (cisplatin-resistant) GCC cell lines. **F.** ChIP-seq data of pan-H3ac occupation in indicated genes of romidepsin / solvent treated TCam-2. Data was illustrated in the 'UCSC Genome Browser'.

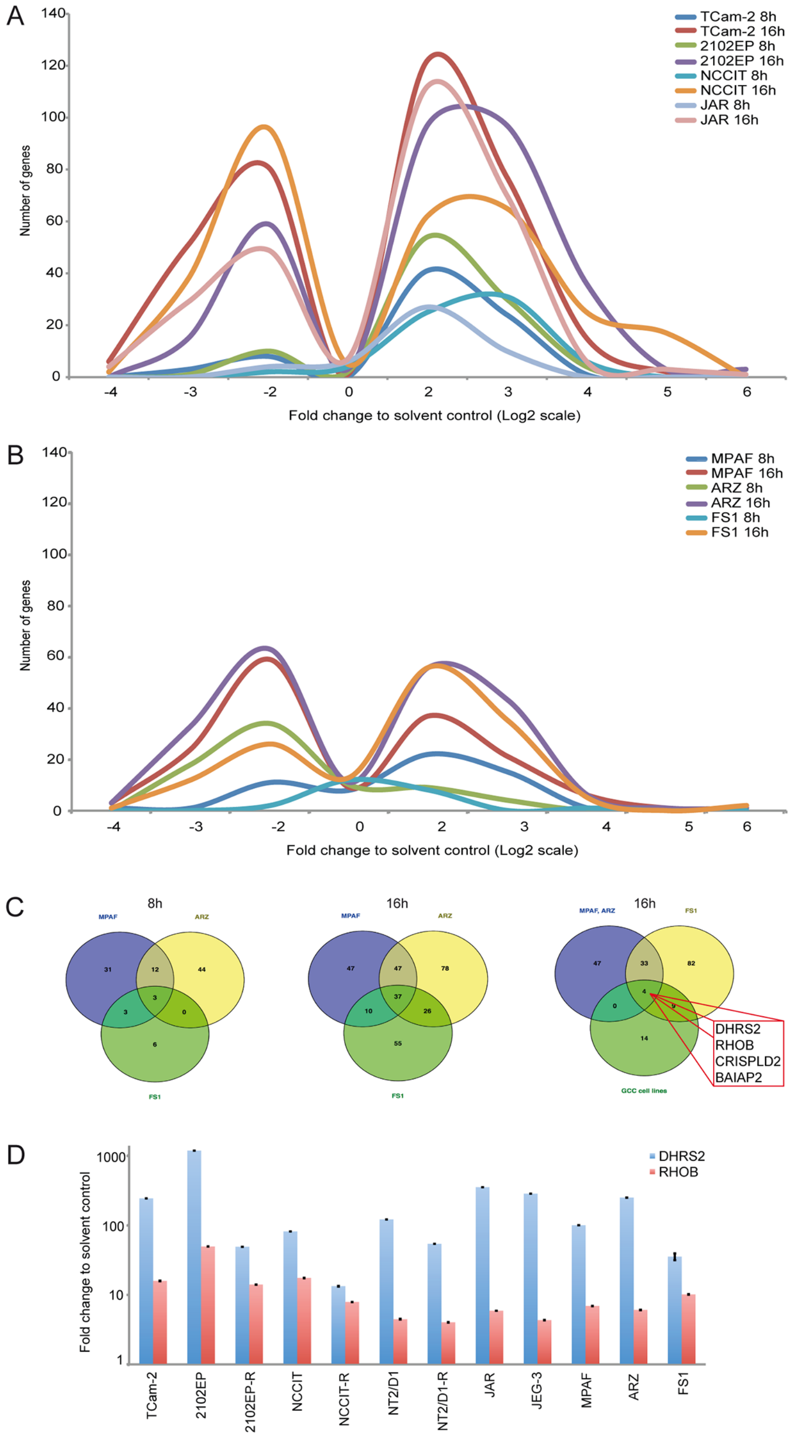

Supplementary Figure S4: A, B. Histogram of genome-wide expression fold change distribution in GCC cell lines (A) and fibroblast as well as FS1 cells (B) 8 and 16h after 10 nM romidepsin treatment based on expression microarray analysis. **C.** Venn diagrams summarizing numbers of commonly expressed genes between MPAF and ARZ after 8 and 16h of romidepsin treatment as well as between GCC cell lines and fibroblasts after 16h based on expression microarray analysis. **D.** qRT-PCR analysis of *DHRS2* and *RHOB* expression in GCC cells and fibroblasts 16h after 10 nM romidepsin treatment.

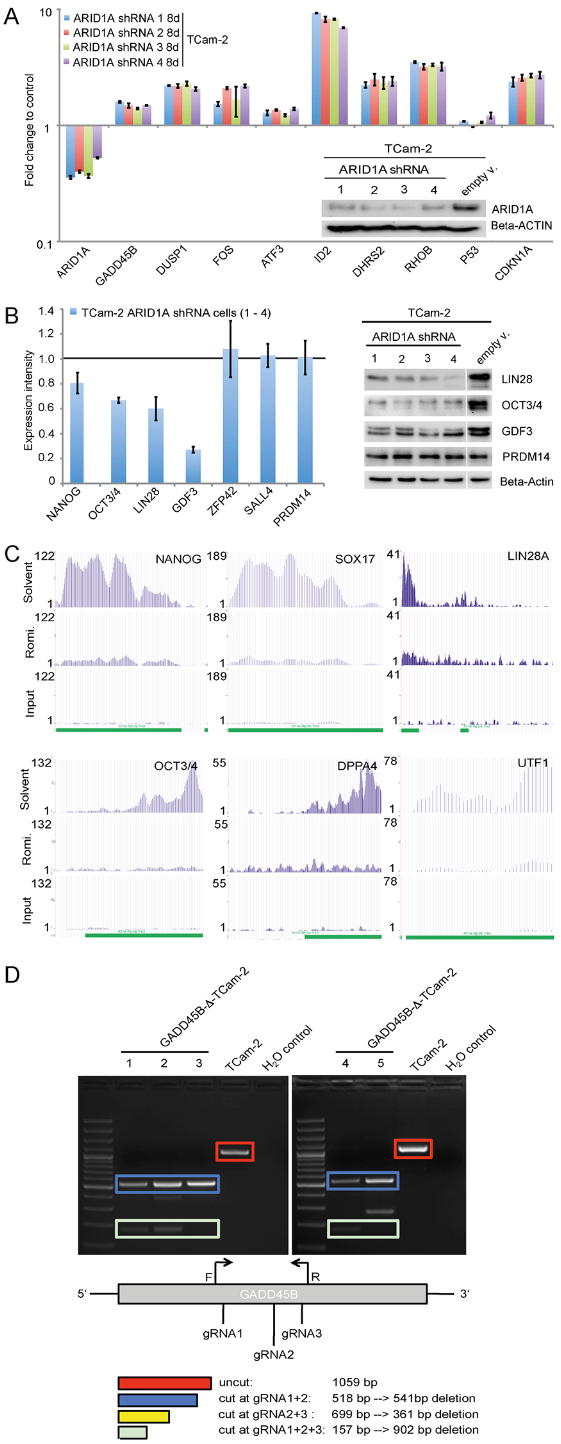

Supplementary Figure S5: A. qRT-PCR and western blot analysis (inlay) of indicated genes after transduction of TCam-2 cells with *ARID1A* shRNA. **B.** qRT-PCR (left) and western blot (right) analysis of expression of pluripotency factors in *ARID1A* shRNA transduced TCam-2 cells. For qRT-PCR, expression levels of TCam-2 cells transduced with the empty vector were set to 1. **C.** ChIP-seq data of pan-H3ac levels across indicated pluripotency-associated genes. Data was illustrasted in the 'UCSC Genome Browser'. **D.** Genotyping PCR analysis of the *GADD45B* locus in TCam-2-ΔGADD45B clones. Parental TCam-2 served as control (TCam-2). No band corresponding to the 'wildtype' *GADD45B* locus (red boxes) was detectable in the TCam-2-ΔGADD45B clones. All TCam-2-ΔGADD45B clones were targeted by all three gRNAs (light green boxes) or gRNA1+2 (blue boxes).

Supplementary Data S1: Expression and ChIP-seq data of romidepsin treated GCC cell lines and fibroblasts

See Supplementary File 1
